# Artesunate improves cryopreserved yak sperm quality by activating SOD1 antioxidant pathway

**DOI:** 10.3389/fvets.2025.1613506

**Published:** 2025-06-27

**Authors:** Yilin Fan, Weibing Lv, Xiaowei Li, Geri Wanma, Jian Li, Xianrong Xiong, Xiaoqiang He, Yanwen Wang, Shi Yin, Wei Fu, Honghong He, Yaqiu Lin, Daoliang Lan, Yan Xiong

**Affiliations:** ^1^Key Laboratory of Qinghai-Tibetan Plateau Animal Genetic Reservation and Utilization, Ministry of Education and Sichuan Province, Southwest Minzu University, Chengdu, China; ^2^Key Laboratory of Animal Science of National Ethnic Affairs Commission of China, Southwest Minzu University, Chengdu, China; ^3^Longri Breeding Stock Farm of Sichuan Province, Dujiangyan, China

**Keywords:** yak sperm, cryopreservation, artesunate, SOD1 protein, antioxidant

## Abstract

**Introduction:**

Sperm damage from oxidative stress is common in semen cryopreservation. Natural antioxidants for frozen semen are largely unexplored. Artesunate (ART), derived from Artemisia annua and used for malaria treatment, shows potential as a frozen semen antioxidant.

**Methods:**

We investigated the effects of various ART concentrations (0.2, 0.5, and 1.0 mM) on yak semen cryopreservation and examined the molecular mechanisms involved.

**Results:**

Post-freezing, sperm quality markedly declined. However, 0.2 mM ART significantly improved sperm motility (both total and progressive) and reduced membrane damage. ART treatment effectively alleviated oxidative stress by reducing ROS levels and MDA content in the sperm while selectively upregulating SOD1 activity and protein levels, which are involved in maintaining DNA integrity and mitochondrial function.

**Conclusion:**

These findings support the hypothesis that ART exerts antioxidant effects via a SOD1-mediated mechanism, highlighting its potential to improve frozen semen quality and offering a novel strategy for cryopreserving sperm in other species.

## Graphical abstract

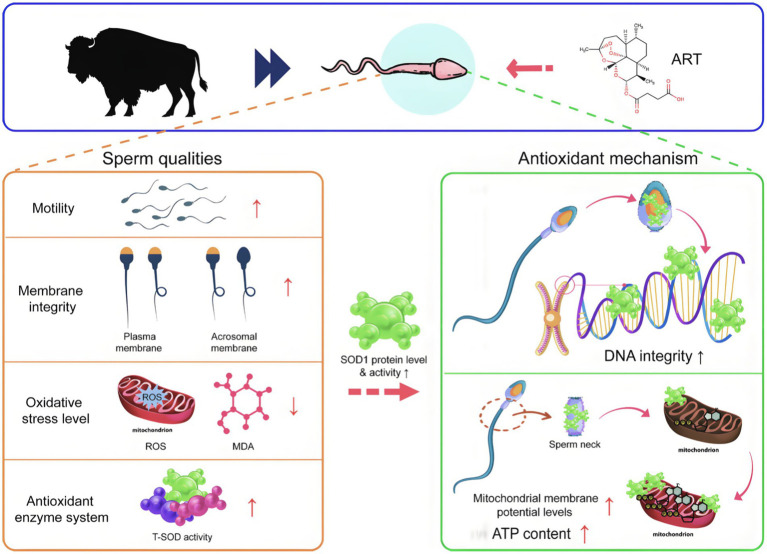

## 1 Introduction

The yak is pivotal to ecological and economic development of Qinghai-Tibetan Plateau, where was referred to as the “third pole” of the world (1, 2). Enhancing yak breeding efficiency and developing robust breeding systems are essential for sustainable industry growth and ecosystem preservation (3). Artificial insemination and assisted reproductive technologies heavily on cryopreserved sperm (4, 5), whose quality directly impacts reproductive success and fertility. Thus, improving cryopreserved sperm quality is a strategic priority for the yak industry and ecosystem protection.

Although sperm cryopreservation techniques have advanced significantly in cattle, post-thaw sperm motility in yaks remains suboptimal for artificial insemination, largely due to freezing and thawing-induced oxidative stress (6), linked to downregulation of the antioxidant enzyme SOD1 and upregulation of NDUFS8 (7), a key ROS generator (8). Semen cryopreservation elevates ROS levels, damaging sperm acrosomes and plasma membranes (9–11). In addition, the high polyunsaturated fatty acid content in sperm membranes renders them particularly vulnerable to ROS-induced lipid peroxidation, leading to malondialdehyde (MDA) formation and oxidative damage (12, 13). Moreover, excessive ROS also causes DNA fragmentation and impairs sperm-egg fusion and fertilization (14–16). Given the diverse damages induced by ROS, utilization of safety and natural antioxidants to mitigate oxidative stress during semen cryopreservation is critical.

Artesunate (ART), derived from the traditional Chinese herb *Artemisia annua*, is FDA-approved for treating severe malaria, including in children, pregnant women, and lactating individuals (17, 18). Beyond its anti-malarial properties, ART exhibits potent antioxidant effects, protecting against oxidative stress in various pathological conditions (19). For instance, ART restored catalase and superoxide dismutase activity in malaria-infected mice (20), and also protected retinal ganglion cells from H_2_O_2_-induced damage at 0.2 mM (21). Despite these findings, the potential of ART as an antioxidant in yak sperm cryopreservation, as well as its underlying mechanisms, have yet to be explored. Thus, this study sought to assess the effects of ART on yak sperm cryopreservation and elucidate its antioxidant mechanisms, providing a scientific basis and new strategy for yak semen cryopreservation.

## 2 Materials and methods

All animal procedures were conducted in accordance with the guidelines of the Animal Care and Ethics Committee of Southwest Minzu University (Approval No. SMU-CAVS-240801001).

### 2.1 Semen collection

The electroejaculation method was used to collect semen from four healthy male yaks (3–5 years old) at the Longri Breeding Stock Farm in Sichuan Province. Initially, the yaks were guided onto a semen collection platform to maintain an upright posture. Then, electroejaculation was applied to the sacral nerves via an electrode rod positioned at the yak’s tail. Generally, the voltage reaches 24 to 30 volts, yak semen can be effectively collected. The voltage is gradually increased by 2 volts at each step. At each voltage level, the output is continuously maintained for 6 to 8 s, followed by a pause of approximately 4 s. This procedure is repeated twice. The semen collection frequency was limited to a maximum of twice a week, with an average semen yield of 5.2 mL per yak. The semen was collected in centrifuge tubes, initially diluted 5–10 fold with Optidyl® diluent (020996, IMV, France) based on its density, and then transported to the laboratory (22–24).

### 2.2 Semen freezing and thawing

Artesunate (ART, A800614, Macklin, Shanghai) was initially dissolved in DMSO (D8371, Solarbio, Beijing) as the stock solution. Subsequently, the Optidyl® diluent was employed to precisely dilute the solution to final concentrations of 0.2 mM, 0.5 mM, and 1 mM. All sperm samples were sealed and subjected to a programmed freezing protocol (TYLDP1, Tianyuanaorui, Beijing; Supplementary Figure 1). After freezing, straws were stored in insulated liquid nitrogen tanks. For thawing, straws were rapidly removed from liquid nitrogen and immersed in a 37°C water bath for 30 s. After thawing, the straws were cut open, and the thawed sperm were expelled for further analysis.

### 2.3 Sperm motility measurement

Sperm samples were divided into five groups, including fresh group, frozen sperm group (NC, 0 mM ART), and three frozen groups respective with 0.2 mM, 0.5 mM and 1.0 mM ART addition. Equal volumes (10 μL) of fresh and frozen–thawed semen were smeared onto slides for testing (Slide: Sail brand, Guangzhou. Cover glass: Citotest, Nantong). The sperm motility parameters were analyzed using AndroVision® CASA software (Minitube, Germany) and a microscope (BX53, Olympus, Japan). The CASA parameters were presented in Supplementary Figure 2. Each sample was analyzed in at least five fields, with over 1,000 sperm counted per sample. Final motility parameters were calculated as the average of the measurements (25).

### 2.4 Membrane structural integrity analysis

Hypotonic swelling solution, used to assess sperm plasma membrane integrity, was prepared by dissolving 0.9 g of fructose and 0.49 g of sodium citrate in 100 mL of distilled water (26). For each group, 100 μL of semen was mixed with 1 mL of hypotonic solution and incubated in a water bath at 37°C for 30 min. Sperm tail morphology was observed under a BX53 microscope (BX53, Olympus, Japan). Sperm exposed to a hypotonic environment will pump water into their plasma membranes. This increases the sperm’s volume-to-surface area ratio, leading to plasma membrane swelling. Intact plasma membranes caused sperm tails to curl due to water absorption, while damaged membranes remained straight (27). At least five random visual fields were selected for each sample, with a total sperm count greater than 1000, and the ratio of curled-tail sperm to the total sperm count was calculated.

The 1:1 (v/v) mixture of 100 μg/mL FITC-PNA (L7381, Sigma, United States) and Hoechst 33258 (C0031, Solarbio, Beijing) was prepared to evaluate the integrity of acrosomal membranes. Each 50 μL of semen was stained with 2.5 μL of the mixture, and smears were prepared on glass slides. The smears were air-dried and then incubated at 37°C for 10 min. Fluorescence images were captured using an Axio Observer microscope (ZEISS, Germany). FITC-PNA binds to acrosomal membrane galactose residues, emitting green fluorescence, while Hoechst 33258 stains nuclei blue (28). At least five random fields were analyzed per sample, with over 1,000 sperm counted. The ratio of intact acrosomal sperm to total sperm was calculated.

### 2.5 ROS level analysis

Equal volumes (200 μL) of fresh and thawed frozen semen were processed following the instructions of the ROS detection kit (S0033S, Beyotime, Shanghai). Fluorescence intensity was measured using a SpectraMax® ID3 microplate reader (Molecular Devices, Shanghai) with excitation and emission wavelengths set at 488 nm and 525 nm, respectively.

### 2.6 Antioxidant indexes analysis

Equal volumes (200 μL) of fresh and thawed frozen semen were processed using assay kits for superoxide dismutase (A001-2, Jiancheng, Nanjing), catalase (A007-1, Jiancheng, Nanjing), glutathione peroxidase (A005-1, Jiancheng, Nanjing), and malondialdehyde (A003-1, Jiancheng, Nanjing). Absorbance values were measured at 550 nm, 405 nm, 412 nm, and 532 nm, respectively, using a SpectraMax® ID3 microplate reader (Molecular Devices, Shanghai). Calculations were performed as per kit instructions. The calculation formulas are as follows:


$$\begin{array}{l} T-\mathrm{SOD}\;(\frac{U}{\mathrm{mL}})=(\text{Control group}\;\mathrm{OD}-\text{Test group}\;\mathrm{OD})/\\ \text{Control group}\;\mathrm{OD}÷50\%\times V\;\text{Total}/\\ V\;\text{Sample}÷\mathrm{Cpr}. \end{array}$$

Note: V_Total_, 3.5 mL. V_Sample_, 0.2 mL. Cpr, Concentration of protein.


$$\begin{array}{l} \mathrm{CAT}\;(\frac{U}{\text{mgprot}})=(\text{Control group}\;\mathrm{OD}-\text{Test group}\;\mathrm{OD})\times 271/\\ V\;\text{Sample}/T/\mathrm{Cpr} \end{array}$$

Note: V_Sample_, 0.05 mL. T, 60 s. Cpr, Concentration of protein.


$$\begin{array}{l} \mathrm{GSH}-\mathrm{PX}=\\ (\begin{array}{l} \text{Nonenzymatic tube}\;\mathrm{OD}-\\ \text{Enzyme tube}\;\mathrm{OD} \end{array})/(\begin{array}{l} \text{Standard tube}\;\mathrm{OD}-\\ \text{Blank tube}\;\mathrm{OD} \end{array})\times \\ \text{Standard substance concentration}/T/\\ \left(\text{Sample volume}\times \text{Sample protein content}\right) \end{array}$$

Note: T, 5 min. Standard substance concentration, 20 μmol/L.


$$\begin{array}{l} \mathrm{MDA}\;\text{content}\;(\frac{\text{nmol}}{\text{mgprot}})=(\text{Test group}\;\mathrm{OD}-\text{Control group}\;\mathrm{OD})/\\ (\text{Standard tube}\;\mathrm{OD}-\text{Blank tube}\;\mathrm{OD})\times \\ \text{Standard substance concentration}÷\\ \text{Sample protein content} \end{array}$$

Note: Standard substance concentration, 10 nmol/mL.

### 2.7 Energy index analysis

Mitochondrial membrane potential was assessed (250 μL semen samples collected from each group), following the instructions of the mitochondrial membrane potential detection kit (C2006, Beyotime, Shanghai). Stained samples were observed and photographed using an Axio Observer fluorescence microscope (ZEISS, Germany), and fluorescence intensity was quantified using a SpectraMax® ID3 microplate reader (Molecular Devices, Shanghai).

ATP content was determined by lysing semen samples in RIPA buffer (G2002, Servicebio, Wuhan) supplemented with protease inhibitors (M5293, AbMole BioScience, United States). ATP levels were measured at 636 nm using an ATP assay kit (A095-1-1, Jiancheng, Nanjing). The calculation formula is as follows:


$$\begin{array}{l} \mathrm{ATP}\;(\frac{\mu \mathrm{mol}}{\text{gprot}})=(\text{Test}\;\mathrm{OD}-\text{Control}\;\mathrm{OD})÷\\ (\text{Standard}\;\mathrm{OD}-\text{Blank}\;\mathrm{OD})\times \\ \text{Standard concentration}÷\mathrm{Cpr} \end{array}$$

Note: Standard concentration, 1000 μmol/L; Cpr, protein concentration.

### 2.8 Western blot

Total protein was extracted from semen using RIPA buffer (G2002, Servicebio, Wuhan). Protein concentration was quantified using a BCA kit (PD101-500T, Oriscience, Chengdu). Proteins were separated by SDS-PAGE, transferred to PVDF membranes, and probed with primary antibodies (SOD1, A0274, ABclonal; GAPDH, GB12002-100, Servicebio) at 1:1000 dilution. Secondary antibodies (Goat Anti-Rabbit IgG HRP, S0001, Affinity; HRP-conjugated Goat Anti-Mouse IgG, SA00001-1, Proteintech) were used at 1:5000. Bands were visualized using an A32747 gel imager (Invitrogen, United States).

### 2.9 Immunofluorescence

The immunofluorescence staining was performed as described (7). In brief, each sample was fixed in 4% PFA for 20 min and washed with PBS. 100 mM glycine was added to the samples and allowed to stained for 10 min before another PBS wash. The samples were then treated with blocking buffer for 1 h, followed by the addition of primary antibody (SOD1, A0274, ABclonal, Wuhan) at a dilution of 1:200 (v/v), and incubated overnight at 4°C. After washing with PBS, the samples were incubated with secondary antibody goat anti-rabbit IgG (A11011, Invitrogen, United States. 1:1000, v/v) and DAPI for 1 h at 4°C. Finally, the samples were observed under a fluorescence microscope (Axio Observer, ZEISS, Germany).

### 2.10 DNA integrity analysis

The 90 μL of semen was taken and placed in a centrifuge tube. The procedure was performed according to the instructions provided by the AO/EB (Acridine Orange/ Ethidium Bromide) staining kit (E607308, Sangon Biotech, Shanghai). The stained samples were then photographed under a fluorescence microscope (Axio Observer, ZEISS, Germany). As described in the instructions, AO has cell permeability and can enter living cells, resulting in uniform green fluorescence in living cells. However, cells with DNA damage exhibit bright green fluorescence due to chromatin clotting and nuclear breakage. For statistical analysis, at least five visual fields were randomly selected from each sample, with a total sperm count of over 1000. The ratio of sperm with intact DNA to the total sperm count was calculated for the selected fields.

### 2.11 Data analysis

Western blot and Immunofluorescence results were analyzed using Image J (version 1.53). Data were presented as Mean ± SEM. Statistical analyses were performed using GraphPad Prism software (version 10) and one-way ANOVA, with *p* < 0.05 considered significant. The correction method for multiple comparisons is Tukey’s multiple comparisons test. Different uppercase letters on the same shoulder in the same column indicated significant extremely significant difference (*p* < 0.01). Different lowercase letters on the shoulder indicated significant differences (*p* < 0.05). Unlabeled letters indicated no significant difference (*p* > 0.05).

## 3 Results

### 3.1 The exogenous addition of artesunate increases the motility of yak frozen–thawed sperm

**Figure 1:**
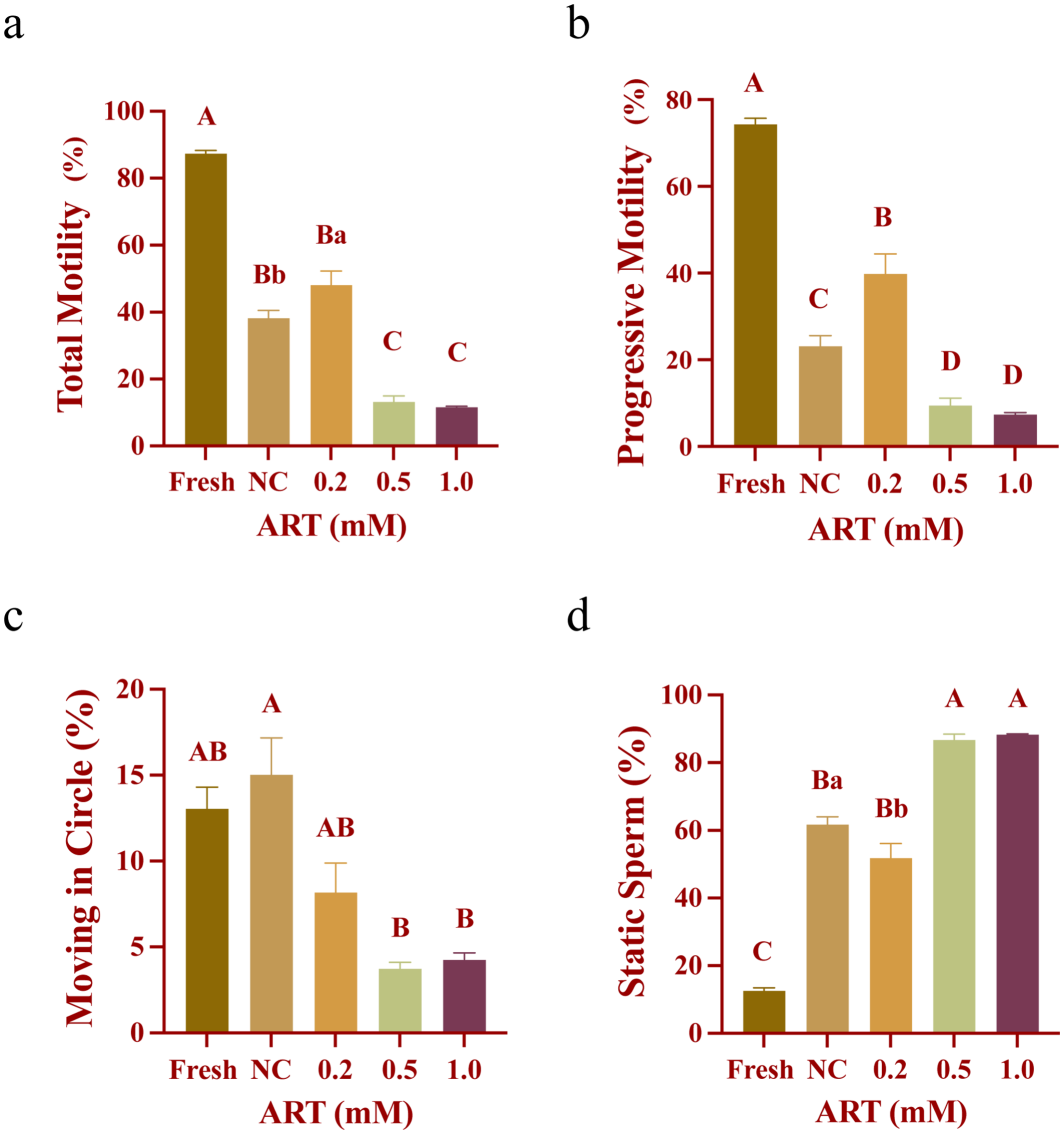
Motility of fresh yak sperm and frozen sperm at different concentrations of artesunate treatment. **(a–d)** The proportion of total motility **(a)**, progressive motility **(b)**, moving in circle **(c)**, static sperm **(d)** in fresh sperm and frozen sperm (NC or different concentrations of artesunate treatment).

Computer-assisted sperm analysis (CASA) was used to evaluate sperm motility among different groups. Fresh sperm exhibited high motility, with total motility exceeding 80% (Figure 1a) and progressive motility around 75% (Figure 1b), significantly higher than the frozen–thawed control group (*p* < 0.01). In contrast, untreated frozen sperm showed markedly reduced motility, with approximately 40% total motility (Figure 1a) and 20% progressive motility (Figure 1b). Notably, 0.2 mM ART restored total and progressive motility to ~40% (Figure 1b; *p* < 0.05; *p* < 0.01), while significantly reducing the proportion of static sperm (Figure 1d; *p* < 0.05). Higher ART concentrations (0.5 mM and 1.0 mM) suppressed sperm motility (Figures 1a–d), potentially due to osmotic imbalance caused by excessive ART. These findings indicate that 0.2 mM ART optimally enhances the motility of yak frozen–thawed sperm.

### 3.2 The exogenous addition of artesunate improves the membrane integrity of yak frozen–thawed sperm

**Figure 2:**
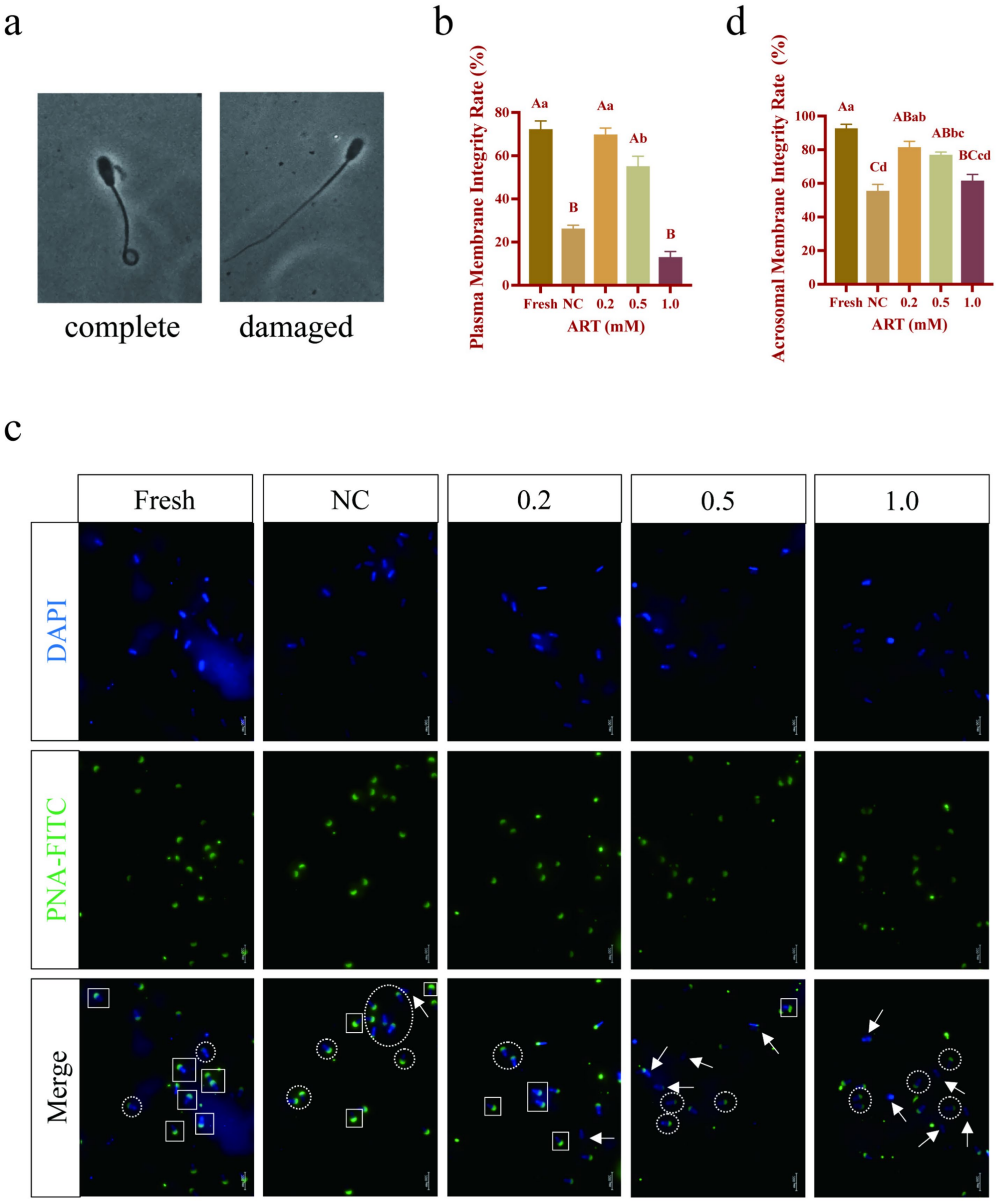
Membrane structural integrity of yak fresh sperm and frozen sperm at different concentrations of artesunate addition. **(a)** Criteria for judging the integrity of sperm plasma membrane. **(b–d)** Plasma membrane integrity statistic **(b)**, acrosomal membrane fluorescence staining **(c)**, acrosomal membrane integrity statistic **(d)** in fresh sperm and frozen sperm (NC or different concentrations of artesunate treatment). White box: acrosomal membrane intact sperm. White circle: acrosomal membrane deficient sperm. White arrow: acrosomal membrane missing sperm. Scale bars 200 μm.

The hypotonic swelling test (HOST) was conducted to evaluate sperm plasma membrane integrity, classifying sperm with curled tails as “intact” and those with straight tails as “damaged” after hypotonic exposure (Figure 2a). Cryopreservation significantly reduced the proportion of sperm with intact plasma membranes (Figure 2b; *p* < 0.01). However, treatment with 0.2 mM and 0.5 mM ART significantly increased this proportion to over 50% (Figure 2b; *p* < 0.01). Acrosomal integrity, evaluated via FITC-PNA staining, revealed higher integrity in fresh sperm and in groups treated with 0.2 mM and 0.5 mM ART, whereas severe acrosomal damage was observed in the control and 1.0 mM ART groups (Figure 2c). Statistical analysis confirmed that the fresh, 0.2 mM, and 0.5 mM ART groups exhibited significantly greater acrosomal integrity than the control group (Figure 2d; *p* < 0.01). These results indicate that appropriate ART concentrations effectively preserve the membrane integrity of frozen–thawed yak sperm.

### 3.3 The exogenous addition of artesunate reduces the oxidative stress level of yak frozen–thawed sperm

**Figure 3:**
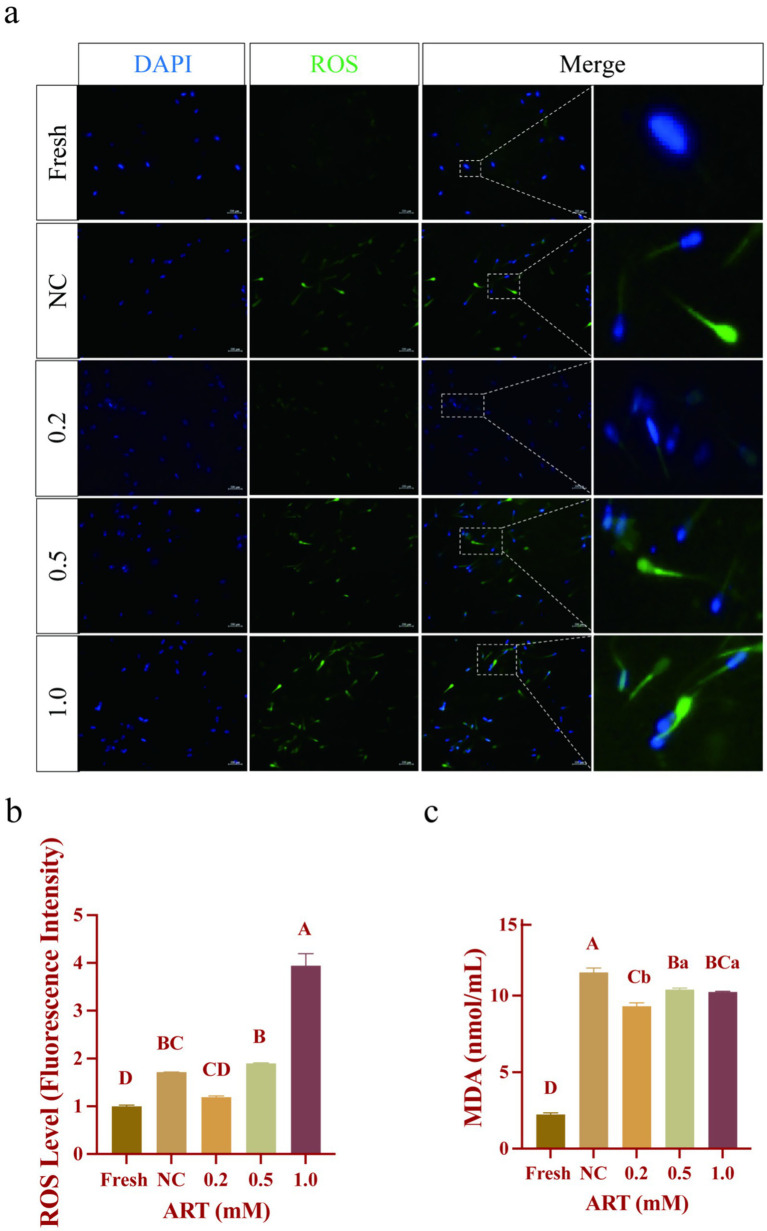
Oxidative stress indexes in yak fresh sperm and frozen sperm at different concentrations of artesunate treatment. **(a–c)** ROS staining **(a)**, ROS fluorescence intensity **(b)**, MDA content **(c)** in fresh sperm and frozen sperm (NC or different concentrations of artesunate treatment). Scale bars 200 μm.

Reactive oxygen species (ROS) and malondialdehyde (MDA) content, which are key indicators of oxidative stress, were measured via fluorescence probe and thiobarbituric acid (TBA) assays, respectively. Fresh sperm exhibited minimal ROS fluorescence, whereas frozen–thawed sperm showed intense signals, particularly in the head and neck regions (Figure 3a). Quantitative analysis revealed that 0.2 mM ART reduced ROS levels compared to frozen–thawed controls (Figure 3b; *p* = 0.0516). Conversely, the 1.0 mM ART group showed the highest ROS fluorescence intensity with ~4 times higher than other groups. Oxidative stress-induced lipid peroxidation, assessed via MDA content, was elevated in the control group (Figure 3c; *p* < 0.01). Notably, 0.2 mM ART treatment markedly decreased MDA levels compared to the control (Figure 3c; *p* < 0.01). These findings indicate that 0.2 mM ART effectively attenuates oxidative stress in frozen–thawed yak sperm by reducing ROS generation and lipid peroxidation.

### 3.4 Artesunate alleviates oxidative stress primarily through maintaining the SOD1 level and activity in the yak frozen–thawed sperm

**Figure 4:**
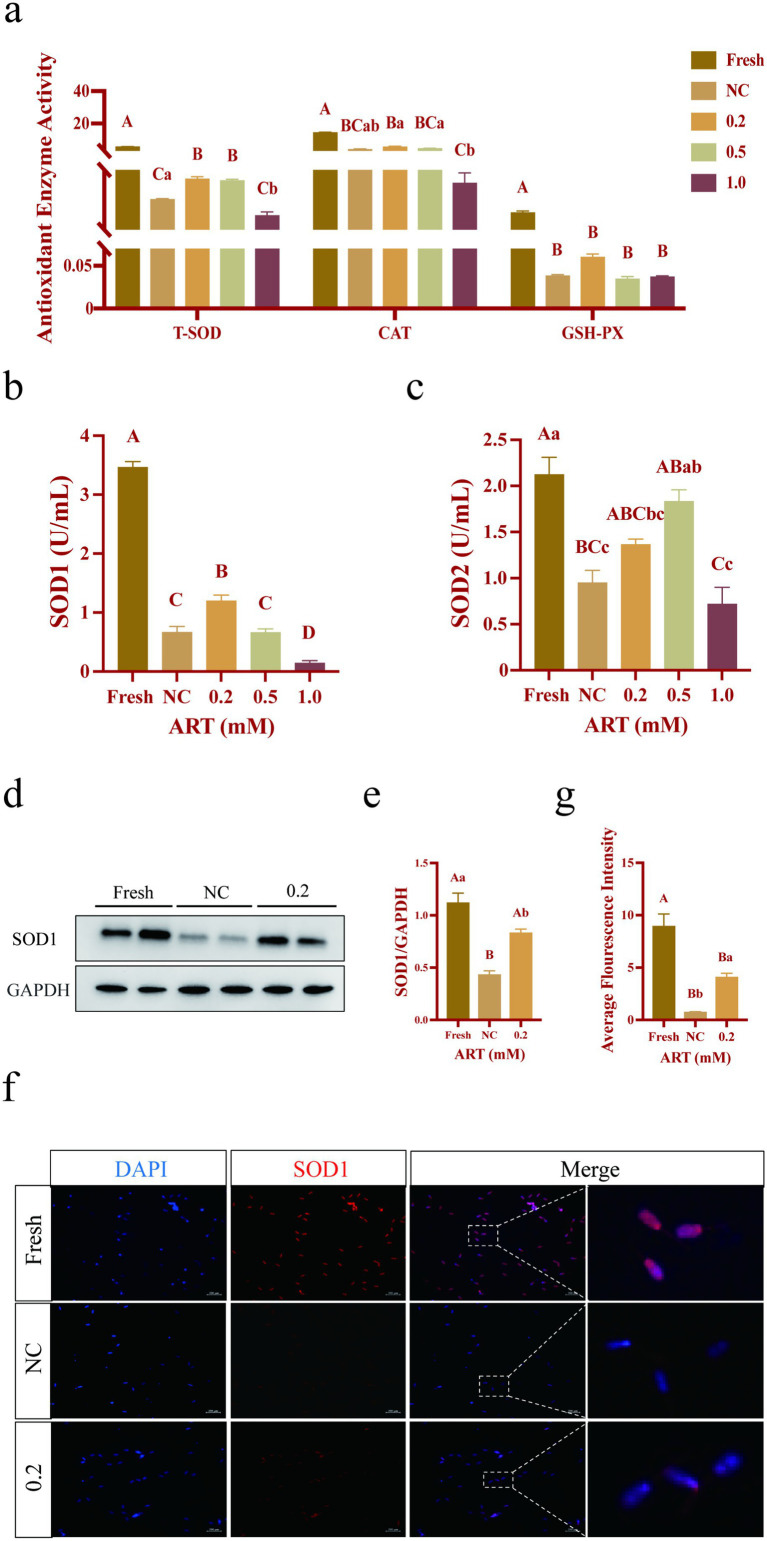
Antioxidant indexes in yak fresh sperm and frozen sperm at different concentrations of artesunate treatment. **(a–e)** Antioxidant enzymes activity **(a)**, SOD1 activity **(b)**, SOD2 activity **(c)**, western blot of SOD1 protein **(d)**, relative gray values of SOD1 protein **(e)**, immunofluorescence staining of SOD1 protein **(f)**, fluorescence intensity of SOD1 protein **(g)** in fresh sperm and frozen sperm (NC or 0.2 mM of artesunate treatment). Scale bars 200 μm.

The enzyme activities of total superoxide dismutase (T-SOD), catalase (CAT) and glutathione peroxidase (GSH-PX) were detected using the colorimetric method, the ammonium molybdate method and the enzymatic reaction method, respectively. As shown in Figure 4a, freezing resulted in significant downregulation of the activities of three enzymes in sperm (*p* < 0.01). Notably, treatment with 0.2 mM ART significantly restored T-SOD activity (Figure 4a; *p* < 0.01), but not that of CAT and GSH-PX (*p* > 0.05). In particular, ART treatment specifically elevated the activity of SOD1 (Figure 4b; *p* < 0.01), while SOD2 activity was not significantly affected by ART (Figure 4c; *p* > 0.05). Western blot and immunofluorescence analysis confirmed a decrease in SOD1 protein in the control group compared to fresh group, and treatment with 0.2 mM ART partially rescued this trend in the sperm (Figures 4d–g; *p* < 0.01; *p* < 0.05). In addition, immunofluorescence imaging revealed that SOD1 is primarily localized in the sperm head and neck regions (Figure 4f). Collectively, these data suggest that ART alleviates oxidative stress primarily through maintaining the SOD1 level.

### 3.5 Artesunate elevates the level of SOD1 to maintain DNA integrity

**Figure 5:**
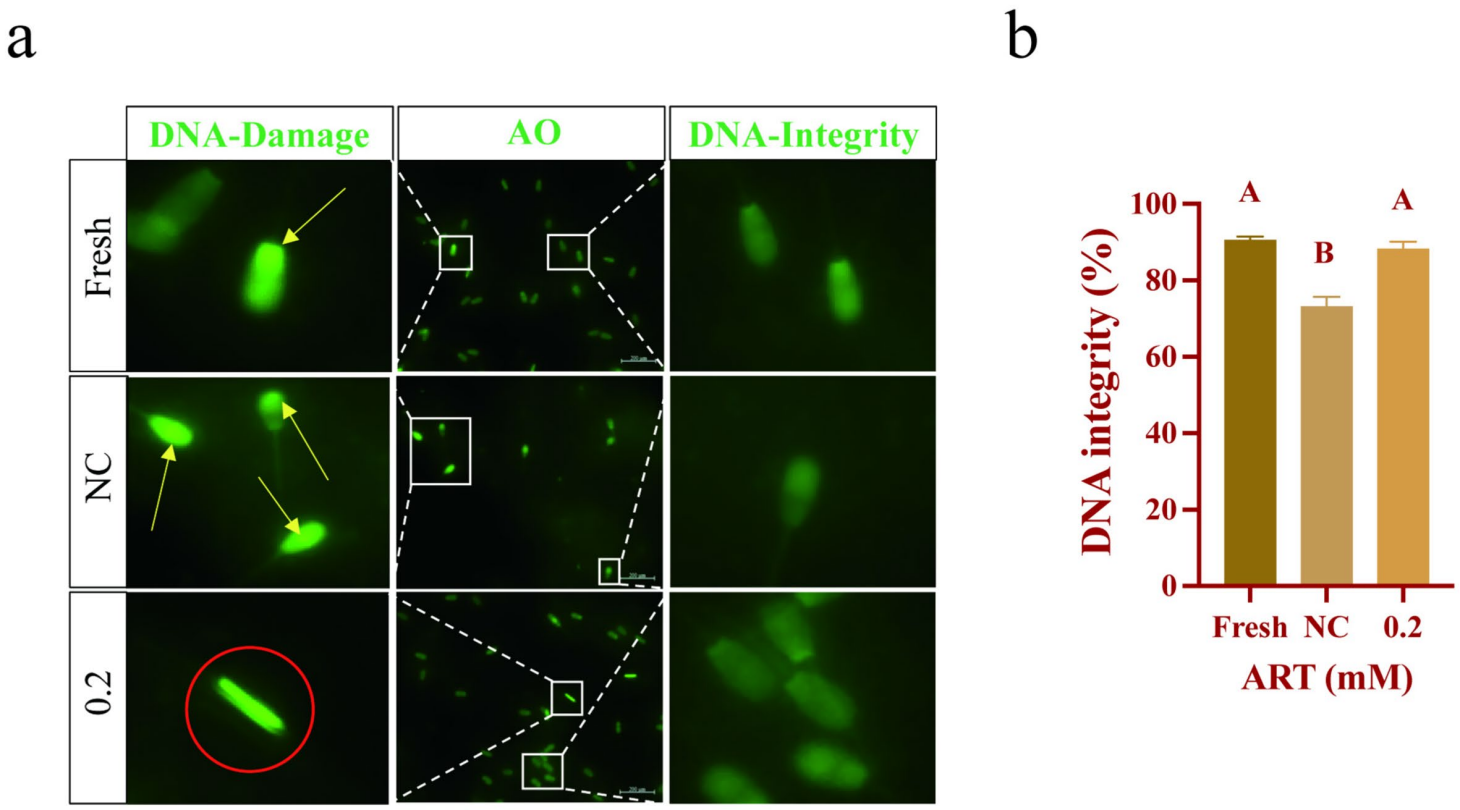
ART regulates SOD1 levels to maintain DNA integrity. **(a,b)** Genomic DNA fluorescence staining **(a)**, genomic DNA integrity statistic analysis **(b)** in fresh sperm and frozen sperm (NC or 0.2 mM of artesunate treatment). Green fluorescence: AO. Yellow arrow: DNA fragments. Red circle: chromatin aggregation. Scale bars 200 μm.

SOD1 is localized in the sperm head, which is implicated in DNA integrity (29), we evaluated it in sperm. AO staining revealed uniform green fluorescence in fresh sperm, whereas frozen sperm exhibited increased bright green fluorescence spots and irregular fluorescence distribution, indicative of DNA damage. ART treatment significantly reduced these fluorescence abnormalities (Figure 5a). Statistical analysis confirmed a significantly lower proportion of sperm with intact DNA in the control group compared to fresh sperm (Figure 5b; *p* < 0.01). Notably, ART treatment markedly reduced frozen–thawed sperm DNA damage (Figure 5b; *p* < 0.01). These findings demonstrate that ART exerts antioxidant effects by upregulating SOD1, thereby preserving DNA integrity.

### 3.6 Artesunate elevates the level of SOD1 to maintain mitochondria function

**Figure 6:**
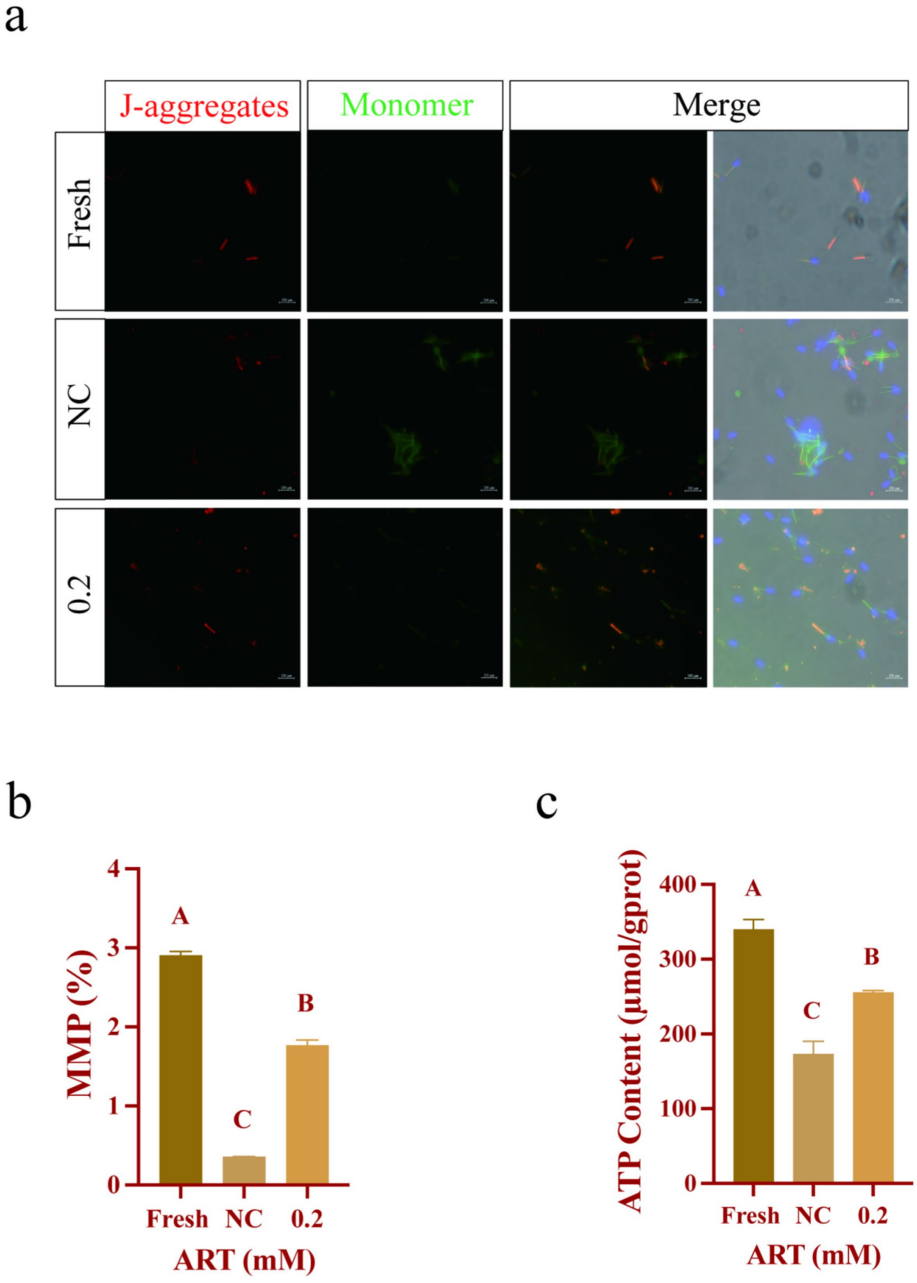
ART regulates SOD1 levels to maintain mitochondrial status and function. **(a–c)** JC-1 staining **(a)**, mitochondrial membrane potential level **(b)**, ATP content **(c)** in fresh sperm and frozen sperm (NC or 0.2 mM of artesunate treatment). Blue fluorescence: DAPI; Red fluorescence: JC-1 aggregates; Green fluorescence: JC-1 monomer. Scale bars 200 μm.

Based on prior evidence that SOD1 is localized to the sperm neck, a region rich in mitochondria (30), we evaluated the mitochondrial status in sperm. JC-1 staining revealed red fluorescence as the dominant signal in the mitochondria of both the fresh group and the ART-treated group (Figure 6a). However, freezing induced a significant increase in green fluorescence within sperm (Figure 6a). Specifically, ART was found to maintain the mitochondrial membrane potential (MMP) in frozen sperm (Figure 6b; *p* < 0.01). Furthermore, ART treatment resulted in a higher ATP content in sperm compared to the NC group (Figure 6c; *p* < 0.01). Collectively, these findings suggest that ART exerts antioxidant effects by upregulating SOD1, ultimately preserving mitochondrial integrity and functionality.

## 4 Discussion

This study investigated the effects of exogenous ART on semen cryopreservation and provided insights into enhancing the efficacy of semen cryopreservation in artificial insemination technology. Three concentrations were designed based on previous studies (21) and our preliminary tests, which significantly improved the motility of yak frozen–thawed sperm upon ART addition. Specifically, the 0.2 mM treatment enhanced the total and progressive motilities of sperm, while higher concentrations (0.5 mM and 1.0 mM) decreased motility, likely due to excessive external additives disrupting the osmotic balance of their environment, as documented in various species (31–34).

In addition, this concentration (0.2 mM) of ART not only effectively maintained membrane integrity, but also reduced oxidative stress, as evidenced by decreasing both ROS levels and MDA content, simultaneously selectively upregulating activity and protein levels of SOD1. The previous studies have shown that cryopreservation significantly affects the expression of oxidative stress-related proteins in yak sperm (7), leading to lipid peroxidation and cell damage (35). Among them, plasma membrane damage is marked by the excessive accumulation of malondialdehyde (MDA) (36, 37).

SOD1 plays a vital role as an antioxidant enzyme by converting superoxide anions into more stable compounds like hydrogen peroxide (H_2_O_2_), thereby reducing oxidative stress levels in sperm (38). The previous research has reported that SOD1 can translocate to the nucleus under elevated ROS production (39), implying its potential importance in preserving genomic stability. Moreover, a recent study demonstrated that the soluble accumulation of SOD1 protein in the nucleus can mitigate DNA damage (29), which is a common consequence of oxidative stress. Comparatively, SOD1 is situated in the mitochondrial intermembrane space and acts to shield mitochondria from oxidative damage (30), which subsequently protects its function and energy production. Our work aligns with previous research indicating that SOD1 is localized in the sperm head and neck regions, where it protects DNA integrity and mitochondrial function. This study demonstrates the multiple effects of ART in reducing oxidative stress damage, which is directly related to reproductive outcomes and the health of potential offspring.

Similar protective effects of SOD1 have been observed in other species, suggesting the broad applicability of ART as a cryoprotectant. In humans, SOD activity in semen is positively correlated with sperm concentration and overall motility, but negatively correlated with sperm DNA fragmentation (40). Similarly, studies in rats have shown that loss of SOD1 leads to impaired daily sperm production and sperm parameters, which in turn may affect fertility potential in adult animals (41). SOD1 was also the most drastically down-regulated differentially expressed protein between fresh and frozen equine sperm (42). These findings collectively suggest that ART, by upregulating SOD1, could serve as a universal cryoprotectant, offering a novel strategy for sperm preservation across species.

In conclusion, our study not only provides a novel strategy for enhancing the quality of cryopreserved yak sperm but also highlights the potential of SOD1-targeted antioxidants like ART in improving reproductive success across species. These findings pave the way for further advancements in assisted reproductive technologies and sperm preservation protocols. In the future, attention should also be focused on the fine regulation mechanism of ART on SOD1, and long-term cryopreserved experiments should be conducted to evaluate the sustained protective effect of ART on sperm quality and offspring health.

## 5 Conclusion

This study elucidates that the addition of 0.2 mM ART significantly preserves the quality of yak frozen–thawed sperm. ART appears to act as an agonist for the SOD1 to active the antioxidant pathway and restore SOD1 protein level and enzyme activity, thereby protecting sperm DNA integrity, enhancing mitochondrial membrane potential, and ultimately improving the quality of frozen sperm. These findings offer critical insights into the functional role of ART in alleviating cryo-damage in yak sperm, thus highlighting its potential as a potent antioxidant for sperm cryopreservation in livestock.

## Data Availability

The raw data supporting the conclusions of this article will be made available by the authors, without undue reservation.
