# Supplementary material for: Artesunate improves cryopreserved yak sperm quality by activating SOD1 antioxidant pathway
Source: Front Vet Sci. 2025 Jun 27;12:1613506. doi: 10.3389/fvets.2025.1613506 (PMC12247533; doi:10.3389/fvets.2025.1613506)
Supplement: Supplementary file 1 [file Data_Sheet_1.pdf]

Supplementary Material

1 Supplementary Figures and Tables

1.1 Supplementary Figures

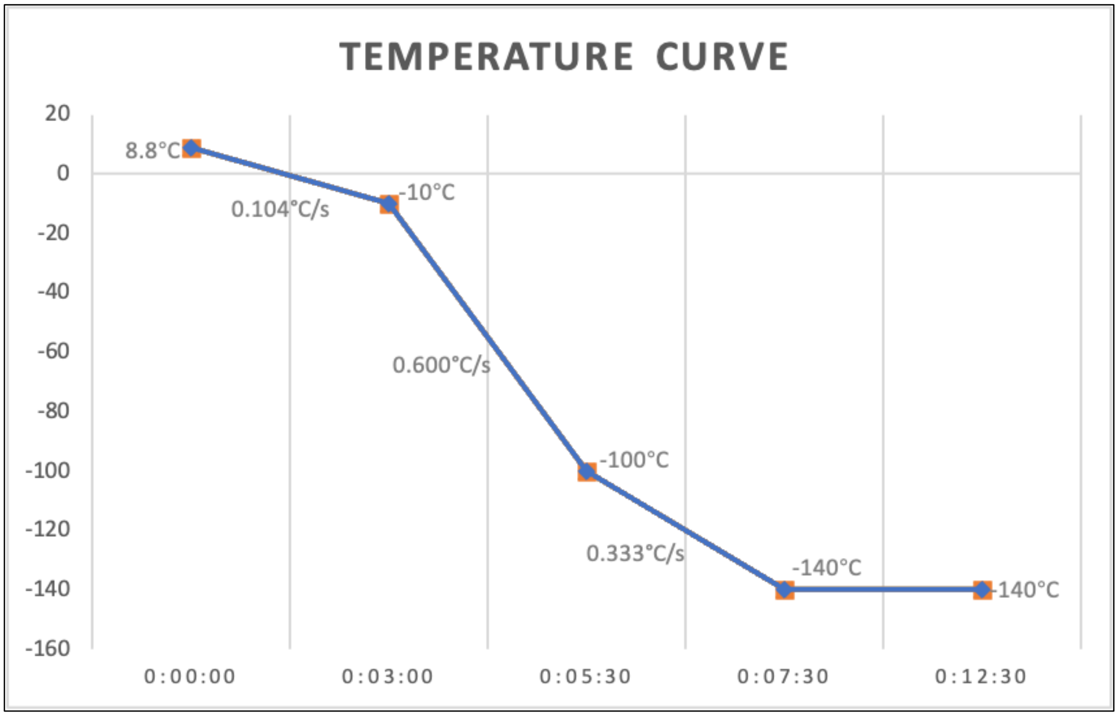

Supplementary Figure 1. Cooling curve of sperm freezing procedure.

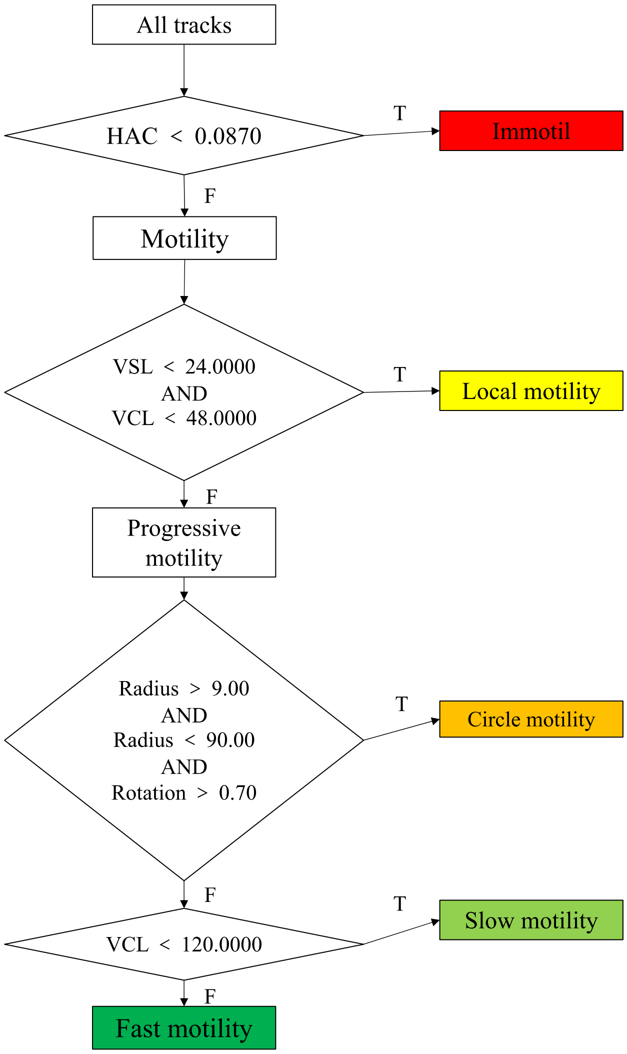

Supplementary Figure 2. Parameters of Computer-aided sperm analysis (CASA).
